# Comparison of Clinical Outcomes in Surgical Patients Subjected to CIPA Nutrition Screening and Treatment versus Standard Care

**DOI:** 10.3390/nu11040889

**Published:** 2019-04-20

**Authors:** José Pablo Suárez-Llanos, Adriá Rosat-Rodrigo, Jennifer García-Niebla, Laura Vallejo-Torres, Irina Delgado-Brito, Miguel A. García-Bello, Francisca Pereyra-García-Castro, Manuel A. Barrera-Gómez

**Affiliations:** 1 Endocrinology and Nutrition Department, University Hospital Nuestra Señora de Candelaria (HUNSC), 38010 Santa Cruz de Tenerife, Spain; francispereyra@telefonica.net; 2 General and Digestive Surgery Department, HUNSC, 38010 Santa Cruz de Tenerife, Spain; roroadri@hotmail.com (A.R.-R.); jeniebla82@hotmail.com (J.G.-N.); mbargom@yahoo.es (M.A.B.-G.); 3 Department of Quantitative Methods in Economics and Management, University of Las Palmas de Gran Canaria, 35001 Las Palmas de Gran Canaria, Spain; laura.vallejotorres@sescs.es; 4 Canary Islands Foundation for Health Research (FUNCANIS), 38109 Santa Cruz de Tenerife, Spain; 5 Health Services Research on Chronic Patients Network (REDISSEC), 38010 Santa Cruz de Tenerife, Spain; 6 Canary Islands Cancer Research Institute (FICIC), 38204 Santa Cruz de Tenerife, Spain; irinadb@hotmail.com; 7 Department of Clinical Epidemiology and Biostatistics, HUNSC; Primary Care Management, 38010 Santa Cruz de Tenerife, Spain; mgarbelx@gobiernodecanarias.org

**Keywords:** general surgery, inpatients, malnutrition, nutrition assessment, length of stay

## Abstract

Malnutrition is prevalent in surgical patients and leads to comorbidities and a poorer postoperative course. There are no studies that compare the clinical outcomes of implementing a nutrition screening tool in surgical patients with standard clinical practice. An open, non-randomized, controlled study was conducted in general and digestive surgical hospitalized patients, who were either assigned to standard clinical care or to nutrition screening using the Control of Food Intake, Protein, and Anthropometry (CIPA) tool and an associated treatment protocol (*n* = 210 and 202, respectively). Length of stay, mortality, readmissions, in-hospital complications, transfers to critical care units, and reinterventions were evaluated. Patients in the CIPA group had a higher Charlson index on admission and underwent more oncological and hepatobiliary-pancreatic surgeries. Although not significant, a shorter mean length of stay was observed in the CIPA group (−1.48 days; *p* < 0.246). There were also fewer cases of exitus (seven vs. one) and fewer transfers to critical care units in this group (*p* = 0.068 for both). No differences were detected in other clinical variables. In conclusion, patients subjected to CIPA nutrition screening and treatment showed better clinical outcomes than those receiving usual clinical care. The results were not statistically significant, possibly due to the heterogeneity across patient groups.

## 1. Introduction

Malnutrition is a prevalent condition in preoperative patients. It is more pronounced in cancer patients and is aggravated by surgery [1,2,3].

Favored by surgical stress and secondary catabolism, malnutrition has been identified as an independent risk factor for immediate postoperative (anastomotic dehiscence, surgical wound infection, sepsis, postoperative ileus, and delayed gastric emptying) as well as medical complications (mainly related to cardiopulmonary complications) [1,2]. The resulting increase in reinterventions, morbidity, and mortality translate into longer hospital stays, more readmissions, and health care costs [4]. In addition, patients with an insidious and prolonged hospital stay may develop sarcopenia, leading to functional deterioration, a worse recovery and long-term survival outcomes [2,4,5,6,7]. It is therefore necessary to detect malnutrition, as a number of studies have shown that treating the malnourished patient improves their clinical prognosis and is cost-effective [8,9,10,11,12,13]. However, there is no “gold-standard” [14], and most European hospitals do not use any of the existing hospital nutrition screening tools [15].

The Control of Food Intake, Protein, and Anthropometry (CIPA) nutrition screening tool has been optimized and validated in several studies, even though it has only been evaluated in nonsurgical patients so far [16,17,18,19,20]. Results are positive when one of the following premises are met in a hospitalized patient: (1) food intake <50% during a 48–72 h period; (2) serum albumin <3 g/dL; (3) body mass index (BMI) <18.5 kg/m^2^ or mid-upper arm circumference (MUAC) ≤22.5 cm (in patients who cannot not be weighed or measured).

In the light of missing published literature, detailed cost-effectiveness and clinical analyses of implementing hospital nutrition screening and related treatments, the present work aimed at contributing novel information to the field [21]. The aim of this study was to compare the clinical outcomes in surgical patients subjected to CIPA nutrition screening and treatment versus standard care. Assuming that such implementation would improve clinical variables and cost-effectiveness, a secondary objective was to promote the use of nutrition screening in other hospitals. Evaluating the economic aspect, we observed that, in addition to improving quality-adjusted life years, the implemented CIPA screening in surgical patients did save costs compared to usual clinical practices [22]. This work presents all the clinical findings in these patients in detail.

## 2. Materials and Methods

### 2.1. Ethical Approval

The Scientific and Ethics Committees of the University Hospital Nuestra Señora de Candelaria (HUNSC) approved the study protocol in January 2015 (PI14/01226-CI-10/15). The study was performed in accordance with Good Clinical Practice standards, applicable local regulatory requirements, and the recommendations of the Declaration of Helsinki, though there were no specific ethical points to be taken into account. Informed consent to participate in the study was obtained from the patients before enrollment.

### 2.2. Trial Design

This study was conducted as a non-randomized controlled trial with two arms: an intervention arm where patients were screened for malnutrition applying the CIPA nutrition screening tool at hospital admission, and a control arm where patients were not screened but diagnosed according to standard clinical practice. This was possible because at that stage CIPA screening had not yet been set up in all the hospital wards (ClinicalTrials.gov NCT02721706). The detailed study protocol and cost-effectiveness analysis have been published elsewhere (the overall study included patients admitted to internal medicine as well as surgical patients). Here, we present the clinical outcomes observed in surgical patients [21,22].

From May 2015 to October 2017, 412 surgical patients were enrolled, 210 in the control group and 202 in the intervention group, according to the hospital wing they were admitted to. Patients aged 18 years and over, who were admitted to the wards of the General and Digestive Surgery Service of the public University Hospital Nuestra Señora de Candelaria and had signed the formal consent to participate, were included in the study. Patients were excluded when: they had been treated with nutrition support prior to CIPA screening or during the equivalent period of time in the control ward; they were transferred from other wards; their expected length of stay was less than 72 h; CIPA screening was unfeasible for any reason; they had received a poor short-term prognosis; they had participated or were participating in distinct research studies; or if they were pregnant.

Sample size calculation was described in the study protocol [21] and was based on the only published comparable study by Kruizenga et al. [23]. Given that our study was performed on the surgical subgroup, power was less than that proposed in the study protocol, attaining 77.8% for a one-sided contrast.

### 2.3. Interventions

CIPA nutrition screening was performed in the patients of the intervention group. Screening outcomes were positive when one of the following premises were met: (1) food intake <50% during a 48–72 h period; (2) serum albumin <3 g/dL; (3) body mass index (BMI) <18.5 kg/m^2^ or mid-upper arm circumference (MUAC) ≤22.5 cm (in patients who could not be weighed or measured).

**Figure 1:**
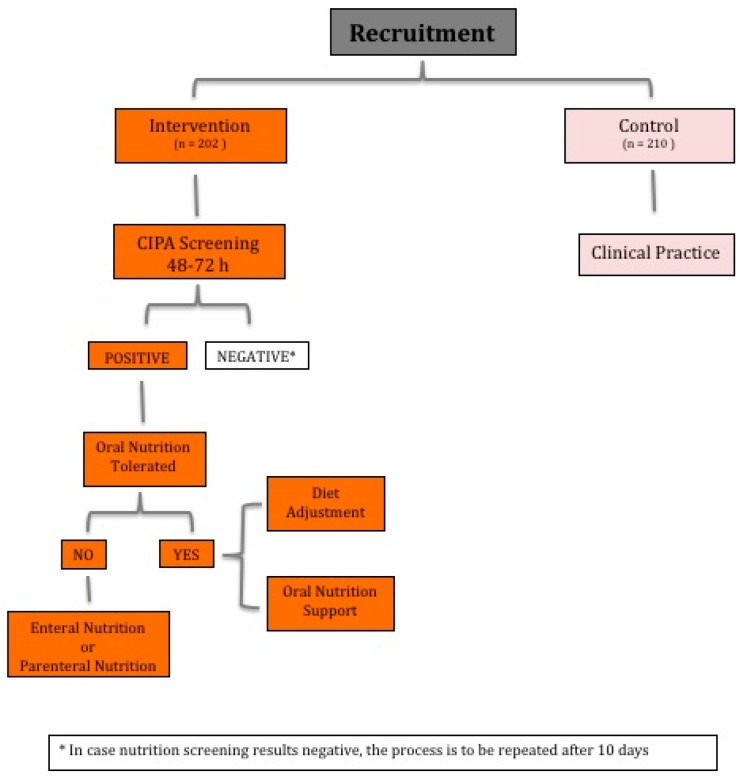
Intervention flow chart. CIPA: Control of Food Intake, Protein, and Anthropometry nutrition screening tool.

In case of a positive screening result, the responsible surgeon was in charge of evaluating the appropriateness of a nutrition treatment. To this end, a protocol was developed for patients able to eat, where an adapted diet was followed up by a dietitian. This diet was based on the patient’s pathology and chewing or swallowing ability and even some personal aversions and preferences. Two additional oral nutrition supplements (ONS) were given (the specific needs of individual patients were not calculated). The individually administered type of ONS depended on the patient’s comorbidities (i.e., diabetes mellitus, renal failure, or oropharyngeal dysphagia) and the positive screening parameters. In cases where there was a positive outcome in food intakes, two bottles of hypercaloric–hyperproteic ONS (approximately 600 kcal and 40 g of proteins per day) were given. In cases where there was a positive result due to the serum albumin concentration, two bottles of normocaloric–hyperproteic ONS (approximately 400 kcal and 40 g of proteins per day) were administered. In cases where the BMI or MUAC determined the result, two bottles of hypercaloric–normoproteic ONS (approximately 600 kcal and 30 g of proteins per day) were applied. If the patient was unable to eat adequately, a suitable artificial nutrition was prescribed (Figure 1). On days three and ten of follow-up, the dietitian assessed adherence to the diet and ONS and a potential necessity of modifying the dose of the prescribed ONS and informed the nurse and the responsible doctor for decision making.

No nutrition screening was performed in the control group, and the responsible surgeons proceeded according to their usual clinical practice. They requested nutrition parameters they considered appropriate (i.e., the patient’s weight, serum proteins, or food intakes) without following any protocol. They then administered specific nutrition treatment depending on the patient’s comorbidities, if they considered that there was a need for it. In order to reduce a potential intervention bias on the patients, doctors responsible for treatment were not made aware of whether patients were enrolled in the study.

After hospital discharge, patients of both groups continued with ONS on an outpatient basis for at least three months according to the responsible doctor’s decision and when financed by the Spanish National Health System (SNHS) [21].

### 2.4. Clinical Outcomes

The main objective was to evaluate the difference in mean lengths of hospital stay between the groups. In addition, the following outcomes were analyzed: readmission rate within 3 months post-discharge; mortality (in-hospital and within 3 months after discharge); incidence of in-hospital clinical complications, evaluated according to the CHADx classification system (Classification of Hospital-Acquired Diagnoses) [24]; transfers to critical care units and need for reinterventions.

In addition, results were evaluated in relation to urgent vs. scheduled surgery, Charlson comorbidity index, the surgery team according to the operated body region (colorectal, gastroesophageal, hepatobiliary-pancreatic, and endocrine), the Clavien–Dindo classification, and the causative pathology (infectious, neoplastic, inflammatory, obstructive, and others).

### 2.5. Statistical Analysis

The individual variables were analyzed in accordance with the intention-to-treat principle. An exploratory and descriptive analysis of the main variables (duration of hospital stay, complications, readmissions, and mortality) was performed with respect to age, sex, Charlson index, EQ-5D-5L quality of life score, surgery team, urgent vs. programmed admission, and the corresponding admitting ward. For hospital stays, a generalized linear model was used with a logit link function. Readmission and mortality were evaluated with a three-month follow-up using logistic regression.

For descriptive analyses of the data, qualitative variables were represented as frequencies and percentages and the continuous variables as means ± standard deviation (SD) or medians in case of notable deviation from normal. Bivariate analyses were performed by means of Student’s *t*-test or the nonparametric Mann–Whitney U test when the quantitative variable was significantly different from normal, the chi-square test for nominal variables, or the Fisher’s exact test, when at least 20% of the expected cells were less than 5. Normal distribution of variables, assumed from visual inspection, was confirmed applying the Kolmogorov–Smirnov test. The STATA v15 program was used.

## 3. Results

There were 210 and 202 patients included in the control and intervention groups, respectively. Fourteen patients refused to participate, eight from the control group and six from the CIPA group. Patients that were excluded from the study because they did not meet inclusion criteria were not quantified.

Table 1 shows the analyzed baseline patient data. There was a larger number of urgent admissions and a larger number of men in the control group, while patients with a more complex clinical picture according to the Charlson comorbidity index prevailed in the CIPA group. Also, more cases of oncological surgery as well as patients with a worse quality of life were found in the CIPA group. Finally, differences were observed in the required surgery. There were significantly more colorectal surgery patients in the control group, while gastroesophageal and hepato-biliopancreatic surgery predominated in the CIPA group.

### 3.1. Nutrition Treatment

In the CIPA group, a total of 45 patients (22%) had a positive screening result. At the discretion of their responsible doctor, none of the patients from the control group were subjected to a food intake control; 32% of these patients were weighed, compared to 79% in the CIPA group (*p* < 0.001). MUAC was only measured in three patients (1%) in the control group vs. 20% in the CIPA group (*p* < 0.001). Finally, serum albumin concentration was determined during the first 3 days of hospitalization in all patients of the CIPA group and in 31% of the control group (*p* < 0.001). In the control group, 33 patients received nutrition treatment (16%) vs. 54 (27%, *p* = 0.006) in the screened group. Mean number of days with nutrition treatment in the CIPA group was higher than in the control group (3.4 ± 12.4 and 3.2 ± 17.6, respectively; *p* = 0.024).

### 3.2. Mean Length of Stay

On multivariate analysis, adjusted for sex, EQ-5D score on admission, Charlson index, patient age, urgent admission, and surgery team, the mean stay in the CIPA group was 1.48 days shorter than in the control group (*p* < 0.246). No statistically significant differences were observed when the type of pathology was added as a covariate. The mean length of stay was shorter with higher EQ-5D scores, and it was shorter in the hepatobiliary-pancreatic surgery team (Table 2).

Although the differences shown in Table 2 seem to be able to affect the results, analyses of a potential effect of interaction between CIPA screening and its associated treatment and the surgical team along with the rest of the covariates did not yield significant results (*p* = 0.59).

### 3.3. Other Clinical Outcomes

With regard to readmissions within the first 3 months post discharge, we did not observe significant differences (*p* = 0.265 on multivariate analysis) between the control (15%) and the intervention groups (19%). The probability increased with increasing Charlson scores (*p* = 0.046) and in urgent admissions (*p* < 0.001).

There were not so many cases of mortality as to achieve significant results within three months post discharge (seven vs. one), although there was a tendency toward lower mortality in the CIPA group (*p* = 0.068).

There were no differences in reinterventions (i.e., 7% in the control and 4% in the CIPA group; *p* = 0.334). Patients who needed urgent admission had a higher risk of reoperation (*p* = 0.003). In accordance with the CHADx index, no significant differences were observed in postoperative in-hospital complications, except for a larger number of overall complications in patients with a worse Charlson score (*p* = 0.002) and quality of life (*p* = 0.001). When complications were classified according to Clavien–Dindo, again no significant differences between the groups (*p* = 0.353) were detected, although a relationship to the patients’ quality of life (*p* = 0.016) and the type of urgent admission (*p* = 0.015) was found. Moreover, we did find that patients in the CIPA group needed fewer transfers to intensive care units (seven vs. one, *p* = 0.068).

## 4. Discussion

There is a lack of information on the benefits of performing screening for malnutrition in hospitalized patients compared to not doing so [23]. The evidence of clinical improvement and reduced costs, which result from nutrition treatment in malnourished, hospitalized patients [7,8,9,13], together with the high prevalence of malnutrition in surgical patients [1,2,3] suggest that performing a simple and cheap nutrition screening associated with a nutrition support protocol with good clinical prognosis would also be cost-effective and generate clinical benefits. This work seeks to shed some light on this information gap by means of the CIPA nutrition screening tool, which meets the aforementioned criteria.

Serum albumin is included as a nutrition marker in the CIPA screening tool, and has demonstrated a predictive value for morbidity, mortality, increased length of stay, and costs [25]. However, it has certain limitations in patients with proteinuria, severe liver disease, or a high level of inflammation. Except for the Glasgow prognostic score, used as an inflammatory marker in oncological patients [26], C-Reactive Protein (CRP) is not used to adjust the albumin as a nutrition marker. Therefore, in accordance with the protocol of this study, this protein was not measured. Likewise, inflammation contributes to anorexia and concomitantly increased nutritional requirements. This is an important point in the development of malnutrition, and albumin can therefore be considered a nutrition marker [25]. Almost all nutrition markers have important limitations (cholesterol, BMI, lymphocytes and even age, which is used in some screening protocols) and most of them can be altered for reasons independent of malnutrition. This is the rationale for why there is no gold standard nutrition screening.

Progressive implementation of the CIPA tool in the hospital center allowed the comparison of a group subjected to screening with one that was not. However, the study could not be randomized through consecutive admissions due to difficulties inherent in clinical practice, as some patient groups tended to enter one ward and not another (there were more colorectal surgery patients in the control group and gastroesophageal and hepatobiliary-pancreatic patients in the intervention group). The same surgical teams supervised patients in both groups. Hence, there was no intervention bias in this sense.

The CIPA tool detected that 22.3% of the patients were malnourished or at risk, and they were treated accordingly. These were somewhat less than observed with the same tool in nonsurgical patients [18]. The proportion of malnutrition in surgical patients given in the literature varies across the types of surgery and whether or not they are oncological. However, our overall data on malnutrition or the risk of suffering from it are within the range described in other works and reviews on more common nutrition screening methods [3,27,28].

Compared to the control group, we observed a lower mean length of hospital stay of one day and a half in the CIPA group (though not significantly different). This occurred in patients with more comorbidities (according to the Charlson index) and a worse perception of their quality of life at the beginning of the study, which conveys the relevance of the finding.

The CIPA group also comprised a higher number of oncological surgeries. The poorer prognosis and complexity of this type of surgery, presenting more post-surgical complications, are well-known and become even worse in malnourished patients [29]. Nonetheless, there were no differences in the number of reinterventions between groups, nor in the number of total complications according to the CHADx nor the Clavien-Dindo classification, the latter being more specific for surgical patients. Moreover, though the patients of this group were more prone to postoperative complications, only one of them needed subsequent transfer to the intensive care unit, whereas seven patients of the control group required this.

With regard to other clinical parameters, neither differences in readmission rates within three months post-discharge nor in deaths were observed, due to these events being rare, although there seemed to be a tendency to reduced mortality in the CIPA group. Though there were no differences between groups in terms of the number of complications, these appeared to be more serious in the control group, as suggested by more transfers to the critical care unit.

Even though more patients in the CIPA group were treated with ONS, a high proportion of the control group (16%) had them as well, which is unusual in clinical practice. The reason might be related with the fact that the same surgeons were responsible for intervention and control group patients. Hence, we might expect an increased awareness of the nutritional aspect of patient care among these doctors. Furthermore, we also found that three times as many patients in the control group underwent surgery in the colorectal zone. The corresponding surgeons were more proactive in pre-surgery nutrition treatment by internal protocol than those on the other sections. This may have contributed to the fact that differences within the analyzed variables were not more pronounced. In fact, we observed that the colorectal surgery group was the only one in which the mean length of stay increased.

## 5. Conclusions

This is the first work to prospectively and concomitantly study and compare clinical variables in in-hospital surgical patients subjected to nutrition screening with the corresponding nutrition treatment with no screening and the performance of usual clinical care. Although patients in the nutrition screening group started in worse clinical conditions, the number of complications was not higher, and the outcomes in the control group seemed even more severe, as there were more transfers to the critical care unit and cases of exitus.

The other clinical variables did not differ between the groups, except for a tendency to a shorter mean stay in the CIPA group, which probably would have been more pronounced or significant if there had been greater homogeneity in the patients of each surgery team, as the patients who received early nutrition support by protocol of the colorectal surgery team predominated in the control group. Moreover, a larger number of enrolled patients would have increased the number of nutrition treatments, thus promoting enhanced clinical performance.

## Figures and Tables

**Table 1:** Baseline sample data.

Category	Control Group (*n* = 210)	CIPA Group (*n* = 202)	*p*
Age at admission	61.4 ± 15.0	62.5 ± 14.4	0.43
Age ≥ 65 years	93 (44%)	95 (47%)	0.576
Sex female	88 (42%)	107 (53%)	0.025
Urgent admission	138 (66%)	103 (51%)	0.002
Surgical pathology			0.065
Infectious	89 (42%)	71 (35%)	0.132
Neoplastic	49 (23%)	70 (35%)	0.011
Inflammatory	19 (9%)	13 (6%)	0.322
Obstructive	20 (9%)	12 (6%)	0.174
Others	33 (16%)	36 (18%)	0.567
Surgery team			<0.001
Colorectal	69 (33%)	23 (11%)	<0.0001
Gastroesophageal	58 (28%)	76 (38%)	0.033
Hepatobiliary-pancreatic	52 (25%)	81 (40%)	0.001
Endocrine	30 (14%)	22 (11%)	0.29
Charlson index	3.0 ± 2.5	3.7 ± 2.7	0.004
EQ-5D-5L score	0.7 ± 0.35	0.58 ± 0.38	<0.001

**Table 2:** Lengths of stay in relation to the surgery team.

Surgical Group	Control Median (percentiles 25–75)	Intervention Median (percentiles 25–75)
Colorectal	6 (5–9)	8 (5–12)
Gastroesophageal	7 (6–10)	7 (6–9)
Hepatobiliary-pancreatic	13 (8–20)	9 (7–14)
Endocrine	6 (4–9)	7 (6–9)
